# Phenolic Constituents of *Lamium album* L. subsp. *album* Flowers: Anatomical, Histochemical, and Phytochemical Study

**DOI:** 10.3390/molecules25246025

**Published:** 2020-12-19

**Authors:** Aneta Sulborska, Agata Konarska, Anna Matysik-Woźniak, Marta Dmitruk, Elżbieta Weryszko-Chmielewska, Agnieszka Skalska-Kamińska, Robert Rejdak

**Affiliations:** 1 Department of Botany and Plant Physiology, University of Life Sciences, Akademicka 15, 20-950 Lublin, Poland; aneta.sulborska@up.lublin.pl (A.S.); elaweryszko@wp.pl (E.W.-C.); 2 Department of General Ophthalmology, Medical University of Lublin, Chmielna 1, 20-079 Lublin, Poland; annawozniak@umlub.pl (A.M.-W.); robert.rejdak@umlub.pl (R.R.); 3 Department of Analytical Chemistry, Medical University of Lublin, Chodźki 4A, 20-093 Lublin, Poland; agnieszkask@wp.pl

**Keywords:** phenolic acids, flavonoids, tannins, secretory trichomes, white nettle corolla

## Abstract

*Flos Lamii albi* has a high biological activity and is widely used in herbal medicine. The aim of the study was to characterize the secretory structures present in *Lamium album* subsp. *album* corolla and the location of phenolic compounds. Additionally, we carried out qualitative phytochemical analyses of flavonoids and phenolic acids. Light, fluorescence, and scanning electron microscopy were used to analyze the structure of the floral organs. The main classes of phenolic compounds and their localization were determined histochemically. Phytochemical analyses were performed with high-performance thin-layer chromatography (HPTLC) and high-performance liquid chromatography (HPLC). Six types of glandular trichomes were found which contained flavonoids, phenolic acids, and tannins. The phytochemical studies demonstrated the presence of caffeic, chlorogenic, ferulic, gallic, p-coumaric, protocatechuic, syringic, gentisic, and vanillic phenolic acids as well as rutoside, isoquercetin, and quercetin flavonoids. The corolla in *L. album* subsp. *album* has antioxidant properties due to the presence of various polyphenols, as shown by the histo- and phytochemical analyses. The distribution and morphology of trichomes and the content of phenolic compounds in the corolla have taxonomic, pharmacognostic, and practical importance, facilitating the identification of the raw material.

## 1. Introduction

The genus *Lamium* L. (f. Lamiaceae) comprises approximately 40 species native to Europe, Asia, and North Africa [1,2]. They are mostly annual or perennial herbaceous plants.

*Lamium album* L. is a 50–100 cm-high perennial herb [2]. It often grows in clusters and covers roadsides, debris, scrubs, and ditches. The species is characteristic of *Lamio-albi Urticenea dioicae* Dengler x Wollert subcl. nov. hoc. loco [3]. Its stem is rectangular in cross section. It has opposite, petiolate, cordate, or ovate leaves with serrated margins [4]. Its bilabiate flowers with a 19–21 mm-long corolla are white or creamy white. Whorls of 5–22 flowers grow from leaf axils in the upper part of the stem [5]. The aboveground plant parts are densely covered with glandular and non-glandular trichomes [4,5].

To date, the morphology of glandular trichomes located on various aboveground parts of some *Lamium* species has been described [6,7,8]; however, corolla trichomes have only been characterized in *L. lycium* [9] and *L. pisidicum* [10]. 

*Lamium album* has been used as a famine food and as alternative nourishment in different countries in Europe, China, and Japan [11]. Its young shoots, leaves, and flowers are edible. They are used for the preparation of teas and raw or cooked food supplements [12]. Supplements with *L. album* extracts are claimed to detoxify the organism [13] (p. 100).

*L. album* flowers contain flavonoids, tannins, phenolic acids, choline, glycosides, saponins, mucilage, iridoids, essential oils, triterpenes, isoscutellarein derivatives, fatty acids, polysaccharides, and amines [2,14,15]. With its considerable diversity of secondary metabolites, the raw material exhibits a high biological activity. Given its anti-inflammatory, antiseptic, bacteriostatic, astringent, mucolytic, and antispasmodic activities, it is widely used in herbal medicine [2,12,15,16]. 

The bioactivity of *L. album* extracts has been evidenced in many scientific investigations. Various pharmacological properties have been determined. The constituents of *L. album* extracts can act as inhibitory factors of virus entry [16], have antioxidant properties [17], and exhibit a protective effect on liver tissue [18] and human fibroblast cell lines [14]. *L. album* can be used as a source of antimicrobial substances. Chloroform, ethanolic, and water extracts have been tested to assess their activity against *E. coli*, *S. aureus*, and *C. albicans* [19]. Extracts of the plant have been reported to reduce the growth of cancer cell lines [20]. Water plant extracts have been found to decrease the inflammation of ocular tissues and the level of human neutrophils [21]. They also exhibit antianemic effects [2,22].

The ecological functions of plant secondary metabolites include plant protection against fungal infection and herbivore attack as well as being attractants to pollinators and seed-dispersing animals and allelopathic agents [23] (pp. 1250–1318), [24] (pp. 229–265).

The whole Lamiaceae family is rich in various active substances [25,26]. The genus *Lamium* is primarily an abundant source of phenolic acids and flavonoids, which are considered as the main groups of substances with multiple biological activities [15,27]. Therefore, these phenolic compounds were chosen for the phytochemical and histochemical tests in our work.

Since the *L. album* corolla is a valuable medicinal raw material and the morphotypes of bioactive compound-producing trichomes located on this organ have not been described, the aim of this study was to examine the content of phenolic compounds and their distribution in glandular and non-glandular trichomes. We also carried out qualitative analysis of flavonoids and phenolic acids in the *L. album* subsp. *album* corolla raw material.

## 2. Results

**Figure 1:**
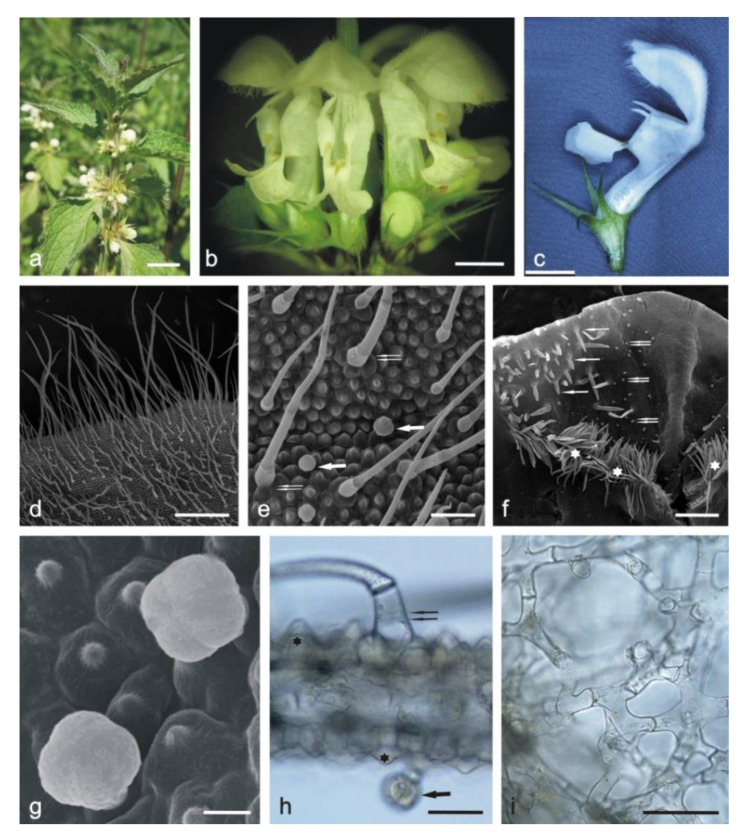
*Lamium album* subsp. *album* flowers and fragments of the corolla. **c**—stereoscopic microscopy (SM), (**d**–**g**)— scanning electron microscopy (SEM), (**h**,**i**)—light microscopy (LM). (**a**) Blooming plants. (**b**) Fragment of an inflorescence. (**c**) Lateral view of the flower. (**d**) Margin of the upper lip with long non-glandular trichomes. (**e**) Various types of trichomes and papillae on the abaxial surface of the upper lip: glandular trichomes (arrows), non-glandular trichomes (double arrows). (**f**) Fragment of the adaxial surface of the corolla tube with unevenly distributed non-glandular conical trichomes (double arrows), glandular trichomes (arrows) and a ring of flattened non-glandular trichomes (stars) located above the ovary. (**g**) Papillae and glandular trichomes with a 4-celled head on the adaxial surface of the upper lip. (**h**) Cross section of the upper lip with visible papillae (asterisks), glandular trichomes (arrow), and a non-glandular trichome (double arrow). (**i**) Loose arrangement of mesophyll cells in the cross section of the upper lip. Scale bars: 1 cm (**a**), 5 mm (**b**), 4 mm (**c**), 500 µm (**d**,**f**,**i**), 100 µm (**e**), 30 µm (**h**), 20 µm (**g**).

The compact *Lamium album* subsp. *album* inflorescences (Figure 1a,b) growing from the axils are composed of flowers with a white two-lipped corolla, which is substantially longer than the calyx. Strongly tapered sepals fuse at an almost half the length (Figure 1c).

### 2.1. Anatomical Analyses

#### 2.1.1. Corolla

**Figure 2:**
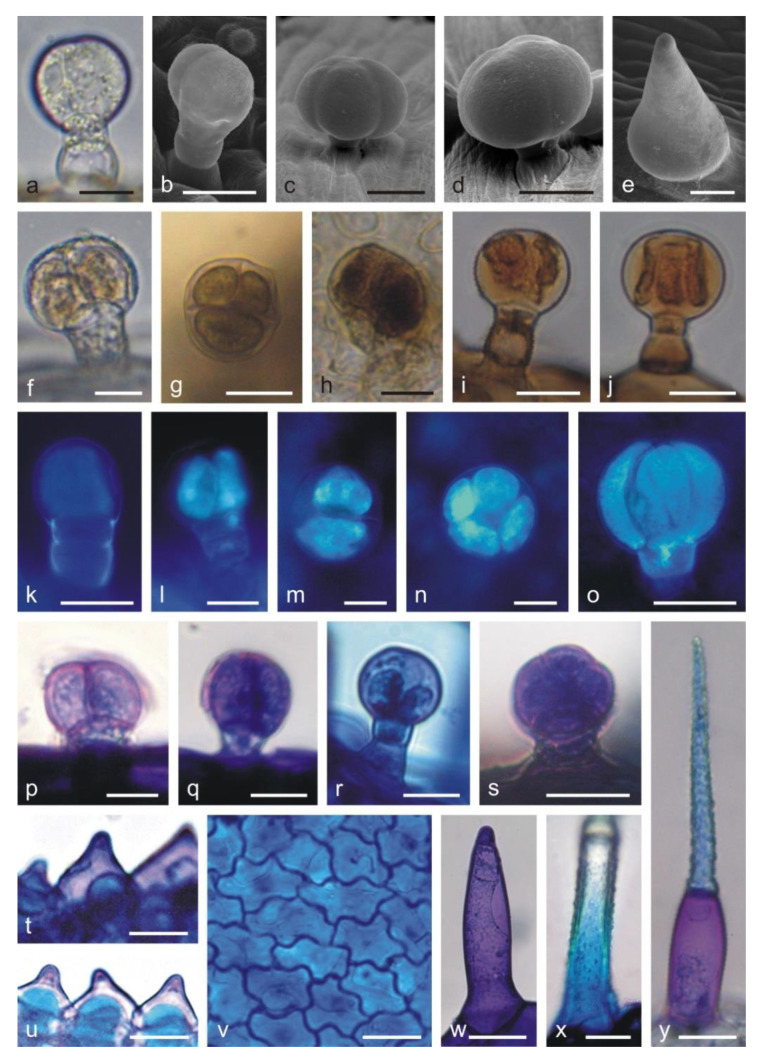
Types of trichomes and papillae on the *L. album* subsp. *album* corolla and the results of histochemical assays. (**a**,**f**) Control trichomes without staining (LM). (**b**–**e**) SEM images of trichomes. (**g**–**j**,**p**–**y**) Histochemical reactions (LM). (**k**–**o**) fluorescence microscopy (FM) images of trichomes. (**a**) Capitate trichome with a 1-celled head. (**b**) Capitate trichome with a 2-celled head. (**c**) Capitate trichome with a 4-celled head. (**d**) Peltate trichome. (**e**) Conical trichome. (**f**) Capitate trichome with a bicellular head and yellow content in the head cells. (**g**,**h**) Phenolic compounds stained black in the heads of trichomes after the application of ferric trichloride. (**g**) Capitate trichome with a 3-celled head. (**h**) Peltate trichome. (**i**,**j**) Brown color of tannins in trichomes stained with potassium dichromate. (**k**–**o**) Blue autofluorescence in different capitate and peltate (**o**) trichomes indicating the presence of phenolic acids. (**p**–**r**) Tannins in capitate trichomes stained blue after Toluidine blue O treatment. (**s**) Peltate trichome stained blue after Toluidine blue O treatment. (**t**–**u**) Phenolic compounds in papillae stained blue after Toluidine blue O treatment. (**v**) Phenolic compounds visible in abaxial epidermis cells of the upper lip after Toluidine blue O treatment. (**w**) Non-glandular conical trichome from the corolla tube stained purple (pectins) after Toluidine blue O treatment. (**x**,**y**) Non-glandular trichomes containing phenolic compounds (blue) visible after Toluidine blue O treatment. Scale bars: 30 µm (**c**,**d**,**h**,**i**,**q**,**r**,**w**,**y**), 20 µm (**a**,**b**,**f**,**g**,**j**,**k**,**o**,**p**,**s**,**t**,**u**,**v**), 10 µm (**e**,**l**,**m**,**n**,**x**).

The adaxial and abaxial surfaces of both corolla lips are covered by different types of trichomes. Long single-layered 1–4 celled non-glandular trichomes cover densely the upper margin on the abaxial surface of the upper lip (Figure 1d) but are less dense on other fragments of the surface. There are substantially smaller glandular trichomes and papillae between non-glandular trichomes (Figure 1e). The lower lip is covered by similar types of trichomes. Inside the corolla tube, there are non-glandular and glandular trichomes. The non-glandular trichomes are long, unicellular, flattened, and pointed. They form a specific ring located above the ovary and the nectary at the bottom of the tube (Figure 1f). Conical trichomes with varying lengths are unique for the corolla tube (Figure 1f and Figure 2e,w). All the glandular trichomes present on the petals are short-stalked (Figure 1g,h). They are composed of a basal cell; a 1–2-celled stalk; and a 1-celled (Figure 2a), 2-celled (Figure 2b,f), 3-celled (Figure 2g), and 4-celled head (Figure 2c,n). The peltate trichomes on the corolla petals have a basal cell, a 1-celled stalk, and a 4–8-celled head (Figure 2d,j,o).

The cross section of the upper lip shows papillae in both the adaxial and abaxial epidermis (Figure 1h). The mesophyll of the petals consists of interconnected branched parenchyma cells arranged loosely with large intercellular spaces (Figure 1i).

#### 2.1.2. Calyx

**Figure 3:**
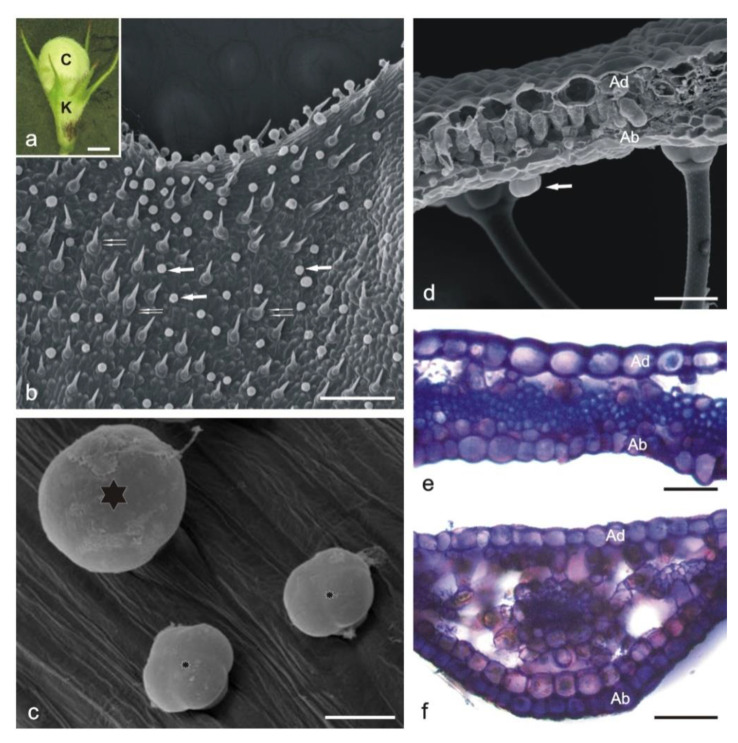
General habit and fragments of the *L. album* subsp. *album* calyx. (**b**–**d**)—SEM; (**e**,**f**)—LM. (**a**) Flower bud with an open calyx. (**b**) Glandular (arrows) and non-glandular (double arrows) trichomes on the abaxial surface of the calyx. (**c**) Peltate trichome (large asterisk) and capitate trichomes (small asterisks) on the abaxial surface of a sepal nerve. (**d**) Cross section of a sepal with fragments of two non-glandular trichomes and a glandular trichome (arrow) on the abaxial surface. (**e**,**f**) Cross sections of sepals (Toluidine blue O staining). Abbreviations: K calyx, C corolla, Ad adaxial epidermis, Ab abaxial epidermis. Scale bars: 2 mm (**a**), 200 µm (**b**), 50 µm (**d**–**f**), 20 µm (**c**).

The calyx consists of five pointed sepals (Figure 3a). In contrast to the adaxial epidermis, the abaxial surface is covered with numerous glandular and non-glandular trichomes (Figure 3b–d). 

**Figure 4:**
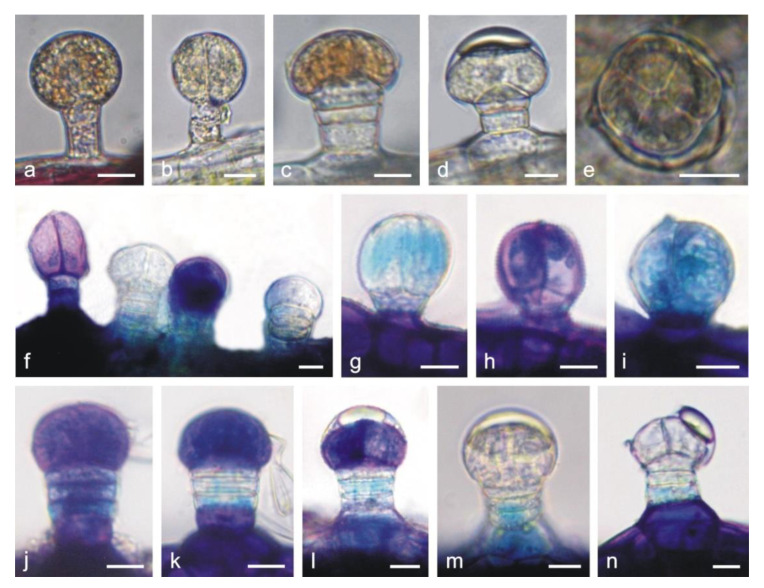
Glandular trichomes present on the *L. album* subsp. *album* calyx in LM. (**a**–**e**) Trichomes from non-stained control preparations. (**f**–**n**) Trichomes stained with Toluidine blue O. (**a**) Capitate trichome with 1-celled head. (**b**) Capitate trichome with 2-celled head. (**c**–**e**) Peltate trichomes at various stages of development. (**f**–**i**) Differently stained (blue and green) capitate trichomes after Toluidine blue O treatment indicating various contents of phenolic compounds. (**j**–**n**) Peltate trichomes with differently stained head cells at different stages of development after Toluidine blue O treatment. In the older trichomes, the secretion is visible in the subcuticular space of the head (**l**,**m**) or on its surface with a ruptured cuticle (**n**). Scale bars: 10 µm (**a**–**n**).

The capitate glandular trichomes are similar to this type of trichomes on the corolla (Figure 4a,b,f). The peltate trichomes on the calyx are longer than on the corolla and consist of a basal cell, a 3–4-celled stalk, and a 4–8-celled head (Figure 4c–e,j–n). The adaxial epidermis cells are larger than those in the abaxial layer and have thicker outer cell walls (Figure 3d,e). The mesophyll consists of palisade parenchyma (1 layer) and sponge parenchyma (2–5 layers), with large intercellular spaces (Figure 3d,f). Only the subepidermal parenchyma cells located below vascular bundles form a compact system (Figure 3f).

The cross sections of the upper zone of the sepals show a layer of mechanical tissue cells with lignified walls and a small diameter in the central part (Figure 3e). Non-glandular trichomes are uniseriate, 2–3-celled, with swollen basal cells (Figure 3d).

#### 2.1.3. Stamens

**Figure 5:**
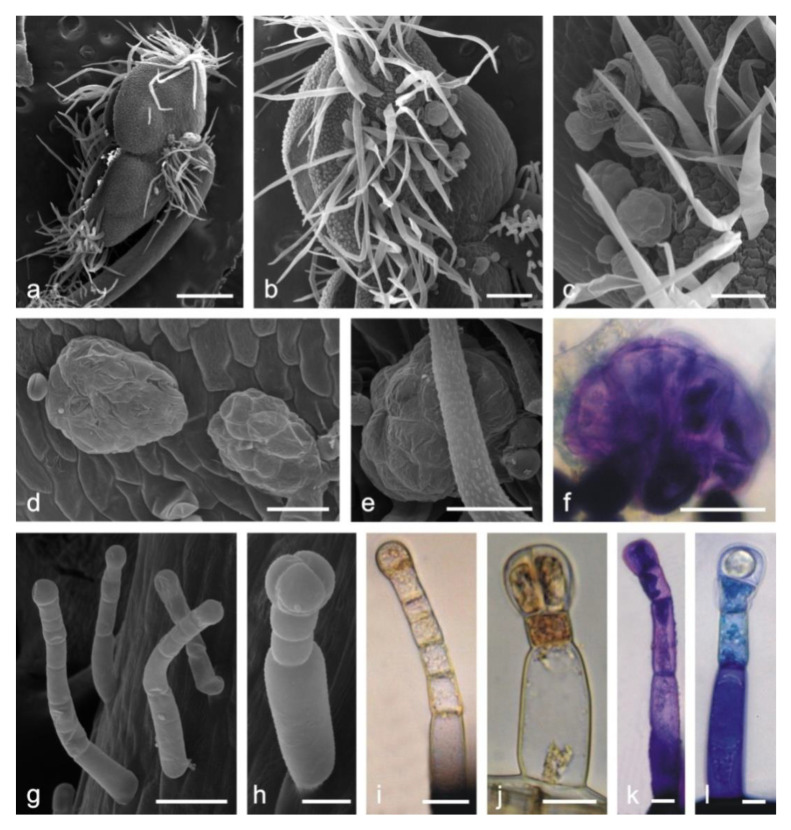
Fragments of *L. album* subsp. *album* stamens with various types of trichomes. (**a**–**e,g**–**h**) SEM; (**f,l**)—LM. (**a**–**c**) Long non-glandular trichomes and multicellular glandular trichomes anthers. (**d**–**f**) Multicellular glandular trichomes. (**g**–**l**) Long-stalked capitate trichomes on filaments. (**f**,**k**,**l**) Tannins stained blue after Toluidine blue O treatment. (**i**,**j**) Tannins stained brown after potassium dichromate treatment. Scale bars: 500 µm (**a**), 200 µm (**b**), 100 µm (**c**), 50 µm (**d**–**g**), 20 µm (**h**–**j**), 10 µm (**k**,**l**).

The trichomes on the stamens have a different structure than those on the calyx and corolla. Some part of the surface of anther in *L. album* subsp. *album* flowers is covered by relatively long one-celled non-glandular trichomes (Figure 5a–c). Additionally, there are large glandular trichomes with a multicellular head and a very short stalk in close proximity to the attachment of the anther and filament (Figure 5c–f). The upper part of filaments bears numerous long capitate trichomes with a 2–5-celled stalk and a mostly rounded 1–4-celled head (Figure 5g–l).

### 2.2. Histochemical Analyses

The application of fluorescence microscopy and histochemical assays facilitated the identification of phenolic compounds—i.e., phenolic acids, tannins, and flavonoids—in the *L. album* subsp. *album* corolla trichomes (Table 1).

In the control preparations, the secretion contained in the head cells of capitate and peltate trichomes was yellowish (Figure 2f). The presence of phenolic compounds was confirmed by the appearance of dark brown staining in the heads of all glandular trichomes (Figure 2g,h) and in papilla-forming epidermis cells of the corolla after the treatment with iron trichloride (not shown). Phenolic acids were detected in the stalk cells of capitate trichomes (Figure 2k and Figure 4b) and in the head cells of all types of capitate and peltate trichomes on the corolla (Figure 5l–o) and in epidermis cells (Figure 4a,b). This was evidenced by blue ultra-violet (UV) autofluorescence, which is characteristic of phenolic acids.

Tannins staining brown in the reaction with potassium dichromate (Figure 2i,j) and deep blue after treatment with Toluidine blue O (Figure 2p–s) were detected in all corolla glandular trichomes. A similar color was recorded in the content and cell walls of papilla epidermis (Figure 2t,u), epidermis (Figure 2v), and non-glandular trichomes (Figure 2x,y), which indicated the presence of phenolic compounds in these cells as well. Additionally, treatment with Toluidine blue O revealed the presence of pectin compounds, which was evidenced by the pink color of the heads of glandular trichomes (Figure 2p,q,s), papillae (Figure 2t,u), and non-glandular trichomes (Figure 2w,y). 

The treatment with Toluidine blue O showed the presence of phenolic compounds staining blue and green in the stalk and head cells in the capitate (Figure 4f–i) and peltate trichomes (Figure 4j–n) on the calyx. The difference in the staining of the trichome cells was probably associated with their different ages. The heads of some trichomes, which probably underwent the final stages of their activity, were not stained by Toluidine blue O, indicating no presence of phenolic compounds (Figure 4f,m,n). The stalk and head cells of the long glandular trichomes covering the stamens contained tannins, which were visualized with the use of potassium dichromate (Figure 5i,j) and Toluidine blue O (Figure 5f,k,l). 

**Figure 6:**
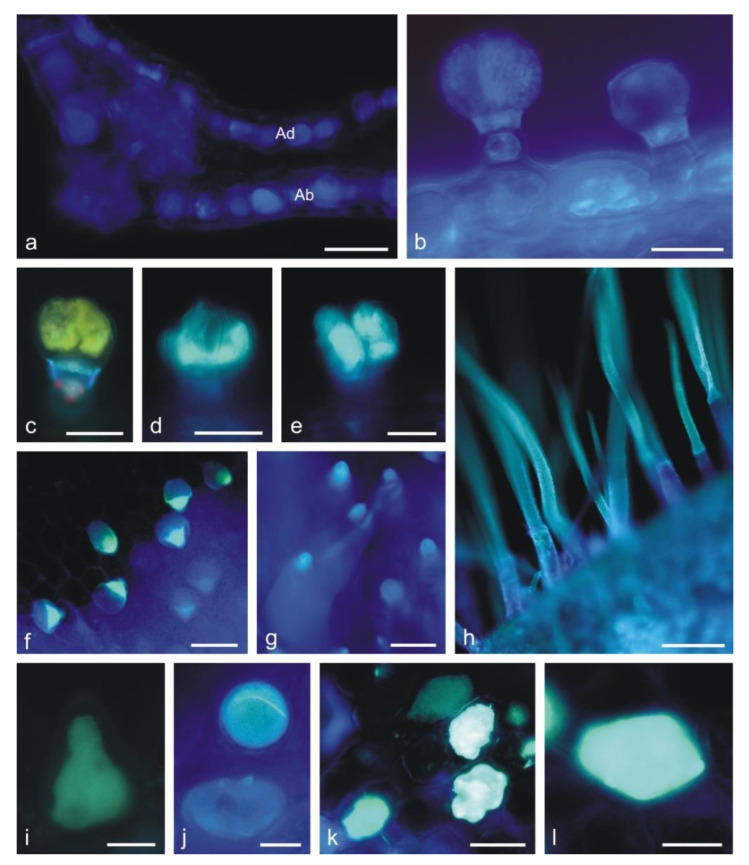
Fragments of corolla petals *L. album* subsp. *album* imaged by a fluorescence microscope. (**a**,**b**) Light blue autofluorescence of phenolic acids (**a**) in epidermis cells in the cross section of the corolla tube, (**b**) in epidermis cells and capitate trichomes. (**c**–**l**) Yellowish secondary fluorescence of flavonoids observed after the application of aluminum trichloride under the Cy 5 filter. (**c**–**e**) Capitate trichomes containing flavonoids. (**f**,**g**) Different-length conical trichomes visible on the adaxial surface of the corolla tube with flavonoids in the apical part. (**h**) Non-glandular trichomes from the upper lip: flavonoid fluorescence in the cell walls. (**i**,**j**) Papillae on the upper lip containing flavonoids. (**k**,**l**) Epidermis cells from the upper lip with flavonoid content. Abbreviation: Ad adaxial epidermis, Ab abaxial epidermis. Scale bars: 100 µm (**g**,**h**), 50 µm (**a**), 30 µm (**b**,**c**,**f**,**k**), 20 µm (**d**,**e**,**l**), 10 µm (**i**,**j**).

The presence of the flavonoids in all types of glandular trichomes on the corolla (Figure 6c–e) and in the non-glandular trichomes (Figure 6f–h), papillae (Figure 6i,j), and epidermis cells (Figure 6k,l) was demonstrated with the use of aluminum trichloride as a fluorochrome, inducing a yellowish secondary fluorescence.

### 2.3. Phytochemical Analyses

**Figure 7:**
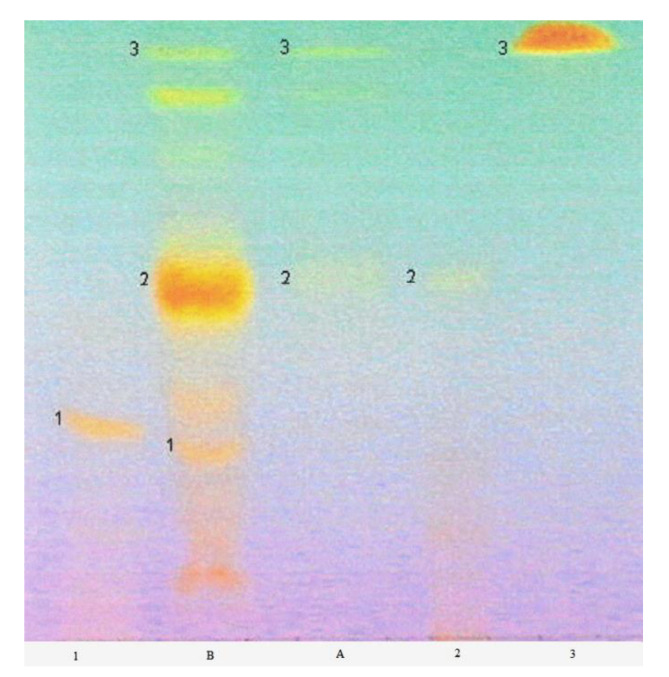
Chromatogram of the separation of flavonoids in methanol extract from *L. album* subsp. *album* flowers with the multiple gradient development technique (MGD). The bands of the standards correspond with the bands of the compounds in the extract: 1—rutoside; 2—isoquercetin; 3—quercetin; A—ethyl acetate extract-excluded from the investigations; B—methanol extract. Visible light, derivatization with the use of a 1% methanolic solution of aluminum chloride. The mobile phase elution program is given in Table 2. Chromatographic plate HPTLC Si 60 F 254.

The flavonoid analysis carried out with the HPTLC method confirmed the presence of rutoside, quercetin, and isoquercetin in the methanol extract of *Lamium album* subsp. *album* flowers (Figure 7). The separation of flavonoids was achieved with the use of the MGD (multiple gradient development) technique. In the visualization of flavonoid compounds (Table S1), aluminum chloride stains the standards blue (Figure S1). The staining of standards of flavonoids with FeCl_3_ is visible as a brown and yellow color in visible light (not shown). 

**Figure 8:**
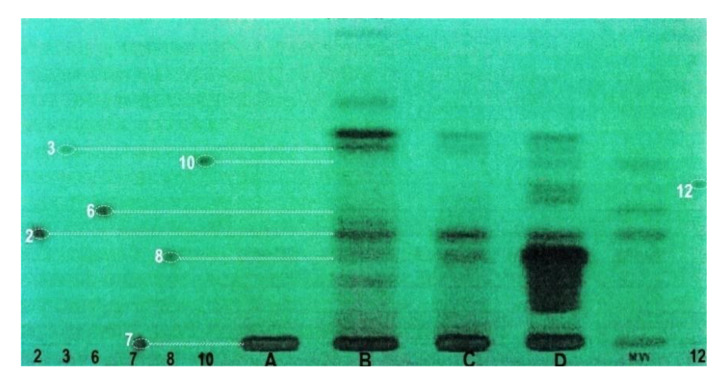
Separation of phenolic acids in methanol extracts of *L. album* subsp. *album* flowers. **A**—ethyl acetate extract-excluded from the investigations; **B**—methanol extract; **C**—acid hydrolysis; **D**—basic hydrolysis; **MW**—mixture of standards; 2—protocatechuic acid (3,4-dihydroxybenzoic); 3—vanillic acid (4-hydroxy-3-metoxybenzoic); 6—syringic acid (4-hydroxy-3,5-dimetoxybenzoic); 7—chlorogenic acid (depside of caffeic and quinic acids); 8—caffeic acid (3,4-dihydroxycinnamic); 10—*p*-coumaric acid (4-hydroxycinnamic); 12—ferulic acid (4-hydroxy-3-metoxycinnamic). Videoscaner Desaga (Germany). Stationary phase—chromatographic plate: Si 60 HPTLC F 254 (Merck). Elution program in Table 3. Wavelength 254 nm.

Three groups of phenolic acids were analyzed in the methanol extract with the HPTLC method: free phenolic acids present in *L*. *album* subsp. *album* flower cells and phenolic acids bound with glycosides and esters. Phenolic acids bound with glycosides were submitted to acid hydrolysis and analyzed afterwards. Phenolic acids bound with esters were released in the basic hydrolysis process (Figure 8). 

The analysis of methanolic extracts in the chromatography experiments was carried out with the use of two methods. First, HPTLC was applied. Protocatechuic, vanillic, and caffeic acids were identified among the free phenolic acids. After acid hydrolysis, the same acids were identified, which implies that these three acids are present in *L*. *album* subsp. *album* flowers in two forms: as free acids and as glycosides. Furthermore, the highest amount of caffeic acid was found in the extract after basic hydrolysis, which suggests that this phenolic acid exists also in an esterified form in the analyzed material. Protocatechuic and ferulic acids were detected as esters. 

**Figure 9:**
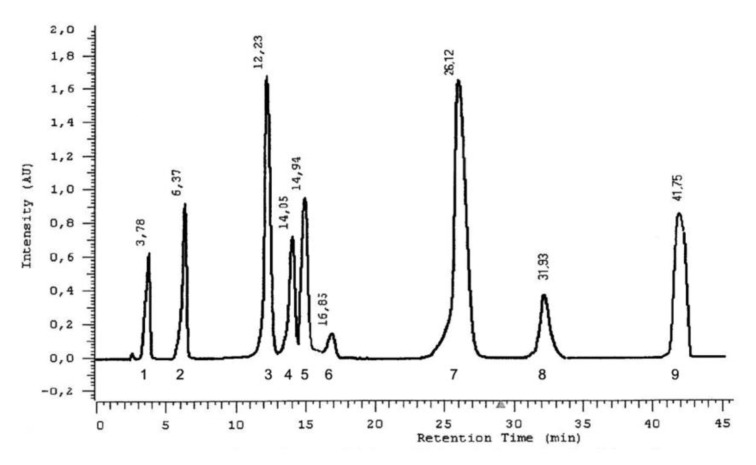
Separation of a mixture of phenolic acid standards in HPLC. Stationary phase—RP-18, mobile phase: water: methanol: formic acid, (75:25:0.5 *v*/*v*). Each compound is characterized by retention time in minutes (given above peaks): (1)—gallic acid (3,4,5-trihydroxybenzoic); (2)—protocatechuic acid (3,4-dihydroxybenzoic); (3)—gentisic acid (2,5-dihydroxybenzoic); (4)—vanillic acid (4-hydroxy-3-metoxybenzoic); (5)—caffeic acid (3,4-dihydroxycinnamic); (6)—syringic acid (4-hydroxy-3,5-dimetoxybenzoic); (7)—*p*-coumaric acid (4-hydroxycinamic); (8)—ferulic acid (4-hydroxy-3-metoxycinnamic); (9)—chlorogenic acid (depside of caffeic and quinic acids). Wavelength 254 nm.

**Figure 10:**
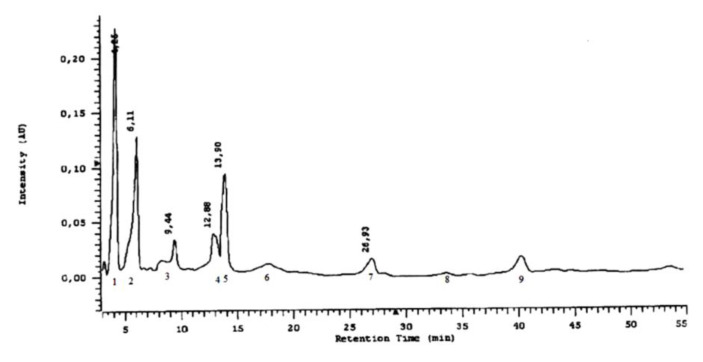
Separation of phenolic acids in methanol extract after basic hydrolysis in HPLC. Stationary phase—RP-18; mobile phase: water: methanol: formic acid (75:25:0.5 *v*/*v*). (1)—gallic acid (3,4,5-trihydroxybenzoic); (2)—protocatechuic acid (3,4-dihydroxybenzoic); peak (3) is not identified; (4)—gentisic acid (2,5-dihydroxybenzoic); (5)—caffeic acid (3,4-dihydroxycinnamic) and vanillic acid (4-hydroxy-3-metoxybenzoic); (6)—syringic acid (4-hydroxy-3,5-dimetoxybenzoic); (7)—*p*-coumaric acid (4-hydroxycinamic); (8)—ferulic acid (4-hydroxy-3-metoxycinnamic); (9)—chlorogenic acid (depside of caffeic and quinic acids). Wavelength 254 nm.

**Figure 11:**
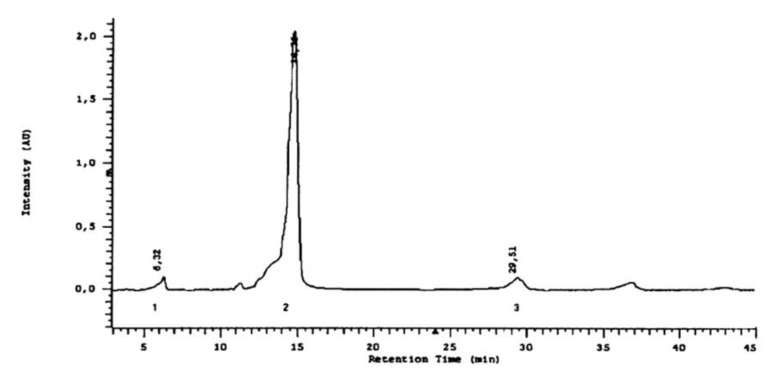
Separation of phenolic acids after the acid hydrolysis of methanolic extract in HPLC. Stationary phase—RP-18; mobile phase: water: methanol: formic acid (75:25:0.5 *v*/*v*). (1)—protocatechuic acid (3,4-dihydroxybenzoic); (2)—caffeic acid (3,4-dihydroxycinnamic); (3)—*p*-coumaric acid (4-hydroxycinamic). Wavelength 254 nm.

The use of the HPLC method provided a more detailed characterization of the forms of phenolic acids in the *L*. *album* subsp. *album* flowers (Figure 9). Nine acids were detected in esterified forms: protocatechuic, vanillic, caffeic, syringic, gallic, gentisic, p-coumaric, and chlorogenic acids, and trace amounts of ferulic acid (Figure 10). Besides the acids detected with the HPTLC method in the extracts after basic hydrolysis, gallic acid was detected by HPLC. This acid exists only in an esterified form in the analyzed flowers. Caffeic and small amounts of protocatechuic and p-coumaric acids were identified with the HPLC method in the extracts after acid hydrolysis (Figure 11). Caffeic acid is the main phenolic acid in a glycosylated form.

## 3. Discussion

### 3.1. Anatomy and Histochemistry

In aromatic plants, phenolic compounds are one of the three classes of natural products, besides fatty acid derivates and terpenes, from which volatile organic compounds originate [28].

Using histochemical assays and fluorescence microscopy, we determined the location of phenolic compounds, including phenolic acids, flavonoids, and tannins, in tissues of the *L. album* subsp. *album* corolla, stamens, and calyx. As shown by the analysis, phenolic compounds were present in all types of glandular trichomes and in the non-glandular trichomes of the *L. album* subsp. *album* corolla and calyx, corolla epidermis cells, papillae, and stamen trichomes.

As shown by the available literature, the types of trichomes on *L. album* subsp. *album* stems, leaves, and calyces have been described to date, but those located on corolla petals have not been studied [8]. We demonstrated differences in the types of trichomes developing on the corolla and the calyx, as well as their diverse morphology. The corolla petals had a greater number of morphotypes of trichomes than the sepals.

Three morphotypes of corolla non-glandular trichomes were distinguished: (i) uniseriate multicellular trichomes formed by 2–4 cells; (ii) one-celled conical trichomes of variable length; and (iii) long, flattened, pointed 1-celled trichomes. In turn, the calyx was covered by only one type of non-glandular trichomes—i.e., unieseriate multicellular hairs (i). This type of trichomes contained tannin-like phenolic compounds, as shown by the reaction with Toluidine blue O. A similar distribution of phenolic compounds in non-glandular trichomes on leaves of another Lamiaceae representative—i.e., *Dracocephalum moldavica*—was reported earlier by Dmitruk et al. [29].

The conical one-celled (ii) trichomes located in the corolla tube as well as some epidermis cells and papillae were found to contain flavonoids emitting secondary yellow fluorescence. The flavonoid content in some leaf epidermis cells and glandular trichomes and in the basal cells of non-glandular trichomes was determined previously in *D. moldavica* [29].

In *L. album* subsp. *album* flowers, the long non-glandular trichomes forming a ring at the bottom of the corolla tube located above the ovary and nectary were an exception, as we did not detect phenolic compounds in these structures.

Peltate and capitate trichomes were distinguished among the glandular hairs present on the calyx and corolla. The peltate trichomes on the calyx epidermis differed from those on the corolla by the greater number of stalk cells (2–3). A similar structure of peltate trichomes containing additional stalk cells was described by other authors on the leaves and calyx of *L. lycium* and *L. pisidicum* [9,10]. In turn, Atalay et al. [8] found short-stalked peltate trichomes with a head formed of 4–8 cells on *L. album* subsp. *album* leaves, which corresponds to the structure of the peltate trichomes on the corolla epidermis observed in this species in the present study. 

The morphology of trichomes covering the *L. album* subsp. *album* corolla corresponds to the data provided by Evert [30] and Bosabalidis and Sawidis [31], who found that peltate trichomes in Lamiaceae consist of a basal cell, a short stalk cell, and a broad head formed of 4–18 secretory cells. However, the peltate trichomes present in the epidermis of the *L. album* subsp. *album* calyx had a greater number of stalk cells, and therefore they do not fit the description. 

The most abundant peltate trichomes on the *L. album* subsp. *album* corolla of flowers analyzed in this study had a four-celled head. Peltate trichomes with a four-celled head were also found in other Lamiaceae species—i.e., *Lamium truncatum* [6], *Ocimum basilicum* [32], *O*. *obovatum* [33], and *Isodon rubescens* [34]. The epidermis of the *L*. *album* subsp. *album* calyx and corolla in our study exhibited four types of short-stalked capitate trichomes with a one-, two-, three- and four-celled head. Our research results differ significantly from the observations reported by Atalay et al. [8], as these authors found only short-stalked and sessile capitate trichomes with a one-celled head on the stems, leaves, and calyx in *L. album* subsp. *album*. The differences may have been related to the younger age of the plants analyzed by these authors, which had not fully developed trichomes with a simpler structure. It is also probable that the ecotypes of *L. album* subsp. *album* growing in Turkey differ in the morphology their trichomes from the ecotypes growing in Poland.

In the present study, long-stalked capitate trichomes with a 1–4-celled head were observed only on stamen filaments in *L. album* subsp. *album*. These trichomes contained phenolic compounds. In turn, Baran and Özdemir [10] reported the presence of similar long-stalked trichomes with a one-celled head on the corolla and filaments in *L*. *pisidicum*.

The histochemical microscopic examinations carried out in the present study demonstrated that phenolic compounds were present not only in the glandular trichomes of the *L. album* subsp. *album* corolla but also in the non-glandular trichomes and epidermis cells. All the glandular trichomes contained phenolic compounds—i.e., phenolic acids, tannins, and flavonoids.

As reported by other authors, phenolic compounds in many Lamiaceae species are contained in glandular trichomes, although these compounds have not been detected in some taxa. In *Stachys*, *Prasium*, *Sideritis*, and *Scutelaria*, most types of glandular trichomes contained phenolic compounds [35,36]. Similar results were obtained in the case of *Dracocephalum* [29], *Isodon* [34], *Satureja* [37], *Melissa* [38], *Marrubium* [39], and *Hyssopus* [40]. In contrast, no phenolic compounds in glandular trichomes were detected in *Ocimum obovatum* [33], *Plectranthus ornatus* [41], and *Salvia aegyptiaca* [42]. 

Flavonoids have also been identified in glandular trichomes in other Lamiaceae species—e.g., in *Salvia officinalis* [43]; 11 species from the genera *Stachys*, *Prasium*, *Sideritis*, and *Scutellaria* [35]; *Dracocephalum moldavica* [29]; *Marrubium vulgare* [39]; and *Thymus quinquecostatus* [44].

The presence of secondary metabolites representing phenolic compounds only in some types of trichomes in many species may confirm the hypothesis postulated by various authors on the different functions of individual types of trichomes [41,43]. 

The results of the present research and the studies cited above are consistent with the findings reported by Richardson [45] on the wide distribution of flavonoids in the Lamiaceae family.

Our investigations have revealed that phenolic compounds are present in different parts of epidermis cells and trichomes. Flavonoids were detected in the cells of the epidermis and papillae and in glandular trichomes located on the *L. album* subsp. *album* corolla. Furthermore, we observed the secondary fluorescence of these compounds in the walls of the non-glandular trichomes. In their previous studies, other authors have reported the presence of flavonoids in the epicuticular waxes, cuticle, and fruit epidermis cells in various plant species [46,47]. Flavonoids were also detected in the protoplasts of epidermis cells and in the basal cells of non-glandular trichomes in *Dracocephalum moldavica* [29].

Tannins were detected in epidermis cell vacuoles, papilla vacuoles, and protoplasts of the glandular trichomes in the studied species. It has been found in other plants that vacuoles in epidermis cells serve as accumulation sites of tannins in the soluble form or in stable complexes with proteins, fats, mucilage, and pectins [48]. Tannin deposits can also accumulate in cell walls, thereby increasing their rigidity [48,49].

We identified phenolic acids in the protoplasts of epidermis cells and in the protoplasts and the cell walls of glandular trichomes. Other authors have detected phenolic acids bound to cell walls in which they serve a structural function—i.e., they enhance wall rigidity [50].

Our anatomical studies and histochemical assays facilitated the localization of phenolic compounds in *L. album* subsp. *album* corollas—i.e., flavonoids and phenolic acids—which were subjected to the qualitative analysis presented in the phytochemical part of the study.

### 3.2. Phytochemistry

Natural plant products give many possibilities of treatment of various illnesses, and their active substances were investigated in many phytochemical studies [51]. Phenolics are one of the principal constituents responsible for the bioactivities of *Lamium* species [27]. It has also been found that phenylpropanoids, flavonoids, iridoids, and phenolic acids are the main compounds of the aerial parts of *L. album* [22,52]. 

The present anatomical studies have shown that flavonoids and phenolic acids are accumulated in the epidermis cells in *L. album* subsp. *album* corolla petals and in the superficial structures of this tissue—i.e., in the outer cell walls, including the cuticle, and in the glandular and non-glandular trichomes. The function of these compounds in the epidermis layer and its structures in the plant consists of protection against UV-B radiation and their role as the first line of defense against pathogens and insects [49]. The flavonoids contained in deeper plant tissues protect them against oxidative damage, and the phenolic acids present in the cell walls are substrates for the synthesis of lignin and suberin [49]. As demonstrated in previous studies, ferulic and p-coumaric acids in plants are located in the cuticle or in glandular and non-glandular trichomes [53].

Phenolic compounds exert a wide spectrum of therapeutic effects on the human organism [49]. Flavonoids and phenolic acids are responsible for the high antioxidant activity of the plant material [27]. The impact of dietary flavonoids on the human organism is associated with a reduced risk of cancer [54], and with the protection of human skin fibroblasts against oxidative stress [55]. Previous studies have shown that quercetin has an inhibitory of effect on cell growth [15]. 

The present phytochemical studies performed with the HPTLC method revealed the presence of three flavonoids, quercetin, isoquercetin, and rutoside, in the methanol extract of *L. album* subsp. *album* flowers. In purified ethanol extracts of underground parts of *L. album*, Pereira et al. [12] detected for the first time the presence of derivatives of flavone isoscutellarein, which constituted one third of the total amount of phenolics. Kaempferol and quercetin derivatives have been identified in 80% ethanol extracts from *L. album* by other authors [56].

Using the HPTLC and HPLC methods, we detected nine phenolic acids—i.e., caffeic acid, chlorogenic acid, ferulic acid, gallic acid, p-coumaric acid, protocatechuic acid, syringic acid, gentisic acid, and vanillic acid—in the methanol extracts after basic hydrolysis of *L. album* subsp. *album* flowers. Caffeic, protocatechuic, and vanillic acids are present in these flowers also as glycosides, and caffeic acid is the main acid released from glycosidic forms. In the methanol and ethyl acetate extracts from *L. album* flowers, Paduch et al. [14] detected six phenolic acids, which were found in the herb as free phenolic acids. Noteworthily, we additionally detected gallic, gentisic, and syringic acid after basic hydrolysis, which was not reported by the authors of the cited study. Caffeic and vanillic acids have essential pharmacological effects in the treatment of depression [57] and periodontal diseases [58].

Comparative studies of phenolic compounds and flavonoids in 15 plant species, including six representatives of the Lamiaceae family, demonstrated the lowest content of these substances in plant shoots with flowers in *L. album* [59]. The authors also found a correlation between the content of phenolic constituents and the antioxidant activity of plants. Other authors compared the phenolic content and antioxidant capacity of *L. album* and *L. maculatum* [60]. Their study showed that *L. album* had a lower content of phenolic compounds and a lower antioxidant capacity than *L. maculatum*. 

The information about the location of flavonoids and phenolic acids in *L. album* subsp. *album* corolla tissues and the qualitative analysis of these constituents confirms the achievement of the goal of the present study. The complementary anatomical and phytochemical studies of the plant material can greatly contribute to the elucidation of the pharmacological effect of the plant raw material.

## 4. Materials and Methods

Flowering *Lamium album* L. subsp. *album* plants intended for the anatomical and phytochemical analysis were collected in the UMCS Botanical Garden in Lublin, SE Poland (51°15′44″ N, 22°30′48″ E). The study was conducted in 2016–2018. The botanical identification of plants was performed using Illustrierte Flora von Mittel Europa [61] and a taxonomic revision of *Lamium* (Lamiaceae) [62]. Voucher specimens of *Lamium album* subsp. *album* supporting this study were deposited in the Herbarium of Department of Botany and Plant Physiology (University of Life Sciences in Lublin, Akademicka 15, 20-950 Lublin, Poland).

Since the corolla is the main medicinal raw material [63], particular attention was focused on this part of the flower. The micromorphology and anatomy as well as the content of phenolic compounds of petals and the structure of trichomes were examined. Corollas of *L. album* subsp. *album* flowers were the material for the phytochemical and histochemical analyses.

The corolla parts (lower lip, upper lip, tube) were observed using stereoscopic (SM), light (LM), fluorescence (FM), and scanning electron microscopy (SEM). Stamens were examined as well, as they constitute the medicinal raw material together with corolla petals. Additionally, sepals were analyzed as a comparative material to check whether the corolla and calyx bear the same types of trichomes. The pistil was not analyzed in the study, as no trichomes were found in the epidermis of this organ. Stamens and sepals were analyzed only with the use of light (LM) and electron scanning (SEM) microscopy.

### 4.1. Histochemical Tests and FM

The content of phenolic compounds in corolla epidermis, its structures, and the stamens was determined using fresh free-hand sections and the following histochemical assays: ferric trichloride for total phenolic compounds [64]; potassium dichromate for tannins [65]; and Toluidine blue O (pH 4) for phenolic compounds, tannins [66,67,68,69], and pectins, the staining of which is limited at their lower content in the cell walls [68,70] (pp. 25–40).

Fluorescence microscopy was employed to detect phenolic acids that exhibit light blue autofluorescence in the presence of UV [71,72]. A FITC (fluorescein isothiocyanate) filter set was used at excitation light of 465–495 nm and a barrier filter was used at a wavelength of 515–555 nm [73]. Additionally, aluminum trichloride was used as a fluorochrome under ultraviolet light with a Cy5 (cyanine dye) filter set (excitation light of 590–650 nm and a barrier filter wavelength 663–738 nm) to determine the presence of flavonoids emitting yellow secondary fluorescence [74]. 

Since some histochemistry and fluorescence techniques do not allow the specific separation of a complex mixture of components [75], we used a relevant number of repetitions (*n* = 10) for each object and each technique and independent parallel reaction to avoid incorrect interpretation. The experiments produced similar positive results 8–9 times. We compared the results with the control. Standard control procedures were carried out simultaneously. Water with glycerine (1:1) slides were used for standard control procedures. Photographs were taken with a Coolpix 4500 (Nikon, Tokyo, Japan) camera coupled to an Eclipse 400 Nikons light microscope (Nikon).

### 4.2. SEM

Fragments of the corolla petals and calyx as well as stamens were fixed in a 4% glutaraldehyde solution in 0.1 M phosphate buffer (pH 7.0). After 12 h incubation at a temperature of 4 °C, the samples were washed in the same buffer four times at 20 min intervals. An ethanol series (30, 50, 70, 90, and 95%) was used to dehydrate the plant material. Next, the samples were immersed three times in absolute alcohol and transferred to acetone. The samples were critical point dried in liquid CO_2_ using Bal-Tec CPD 030 (Balzers, Liechtenstein). 

Dried fragments of the examined floral parts were glued onto stubs with the use of a double-sided carbon tape. The samples were coated with a 10 μm gold layer using a Polaron SC 7640 sputter coater (Emitech). A scanning electron microscope TESCAN/VEGA LMU (Tescan) at an accelerating voltage of 30 kV was used for the examination of the material.

### 4.3. Phytochemical Analyses

#### 4.3.1. Extract Preparation and Purification

Flowers of *Lamium album* subsp. *album* without calyces were collected in summer 2017 (June to September), dried at room temperature without the direct sunlight, and stored at a temperature of 17 °C. Two groups of bioactive compounds were analyzed: flavonoids and phenolic acids. The liquid-liquid extraction process was used in the extract preparation.

To prepare the extract for the analysis of flavonoids, 10 g of *L. album* subsp. album flowers were taken and exhaustively extracted with methanol. Three-step extraction, with three portions of methanol (50 mL), was conducted in an ultrasonic bath (Sonorex Typ RK 102 HB, Bandelin, Berlin, Germany) according to the method described before [76]. The extract was concentrated to 20 mL and used in HPTLC experiments.

Purification and fractionation of phenolic acids was conducted to use the samples in HPTLC and HPLC experiments. After exhaustive extraction of plant material (10 g) with three portions of methanol in ultrasonic bath, the extracts were pooled, evaporated, dissolved in hot distilled water (100 mL), and purified with petroleum ether [77]. The extract of free phenolic acids was obtained after extraction with diethyl ether. To analyze the phenolic acids bound with glycosides and in combination with esters, the hydrolysis process was conducted according to the method described before [78]. The water fraction remaining after the extraction of free phenolic acids was halved (two portion of 50 mL volume) and subjected to acid and basic hydrolysis. The extract of phenolic acids from esterified conjugations was obtained after basic hydrolysis at pH 12. The extract of phenolic acids unbounded from glycosides was obtained in acid hydrolysis (pH 2). In both cases, unbounded phenolic acids were extracted with the use of diethyl ether. Ether fractions were evaporated to dryness and washed with methanol (5 mL) to obtain extracts for use in the HPTLC and HPLC experiments.

#### 4.3.2. Planar Chromatography Experiments

Chromatography experiments were performed on 100 × 100 mm glass plates percolated with a 0.25 mm layer of silica-HPTLC Kieselgel Si 60 with a fluorescent indicator (Merck, Darmstad, Germany). Before use, the plates were washed with methanol and acetone and dried for five minutes at 105 °C for activation. During the experiments, the plates were developed in horizontal Teflon DS chambers (Chromdes, Lublin, Poland). The distance was 90 mm. Before the development, the plates were conditioned for 15 min above the mobile phase. All the solvents used in the planar chromatography experiments were of pro-analytical grade and were purchased from Polish Reagents (POCh, Gliwice, Poland). Techniques of isocratic elution and MGD (multiple gradient development) techniques were used. In the MGD technique, the plate was developed several times (2–9 times). The migration of the mobile phase was dependent on the composition of eluents—the elution strength of the mobile phase was adjusted to the type of the sample [79]. After each development step, the eluent was evaporated from the chromatographic plates.

#### 4.3.3. Analysis of Flavonoids 

Flavonoid standards were purchased from Sigma (St. Louis. MO, USA) and prepared as 0.1% solutions in methanol. The best separation of flavonoid standards was obtained with the use of a mobile phase consisting of toluene, hexane, formic acid, ethyl acetate, and methanol (Table 2) 

#### 4.3.4. Visualization of Flavonoids

Flavonoids have an ability to form complex compounds with metal ions. This was used in the derivatization of chromatographic plates with ethanol solutions of aluminum chloride and FeCl_3_ (Figure S1). A total of 2 µL of flavonoid standards were applied with the use of a Hammilton syringe.

#### 4.3.5. Analysis of Phenolic Acids

Phenolic acid standards were purchased from Sigma (St. Louis. MO, USA) and prepared as 0.1% solutions in methanol. They are specified in the description of in Figure 8 and Figure 9. 

Many mobile phases were tested in the HPTLC analyses of phenolic acids. The best separation of phenolic acid standards was achieved with the use of two mobile phases in the gradient multiple program of chromatogram development (Table 3), distance 90 mm. Plates were dried at room temperature for approximately 20 min in a stream of air after each step of development.

The best separation of standards and application of chosen mobile phases for analysis of three extracts of *L. album* subsp. *album* flowers are shown in Figure 8. The plates were conditioned with the use of the mobile phase from the first step of development. The plate was photographed with the use of a Videoscanner (Desaga, Germany) at λ = 254 nm.

#### 4.3.6. Chromatographic HPLC Experiments

All the solvents used in the HPLC method were pro-HPLC grade and were purchased from Polish Reagents (POCh, Gliwice, Poland). Standard solutions of phenolic acids were prepared as 0.01% methanol solutions, as presented in Figure 9. The separation of phenolic acid in methanol extract was carried out after acid and basic hydrolysis. The investigated compounds were identified by retention times and by a comparison of the absorption maxima of the standards. Each phenolic acid in the HPLC analysis is characterized by retention time in minutes and by absorption maxima in the range 200–400 nm (Table 4).

The HPLC analyses were carried out with the use of a Liquid Chromatograph La-Chrom—Merck with a diode array detector DAD (L-7455), pump (L-7100), degasser (L-7612), thermostat (L-7250), rheodyne injector (20 µL), and Zorbax steel column SB-C 18, dimensions 250 mm × 4.6 mm with a 5 µm grain stationary phase. The investigations were conducted in reverse mode phases. The stationary phase was silica gel with octadecyl groups (RP-18). The mobile phase consisted of methanol and water (25:75, *v*/*v*), with a 0.25% addition of 40% formic acid. The flow rate was 1 mL/min. The measurements were conducted at 25 °C, and the injection volume was 20 µL. The wavelength range was 200–400 nm.

## Figures and Tables

**Table 1:** Histochemical reactions of *Lamium album* subsp. *album* corolla trichomes.

Target Compounds	Stain Reagent	Reaction Color	Trichomes			Papillae	Epidermis Cells
			Peltate	Capitate	Non-Glandular		
General phenolic compounds	FeCl_3_	black, brown	+	+	nd	+	+
	Toluidine blue O	green or blue	+	+	+	+	+
Tannins	Potassium dichromate	brown	+	+	nd	+	-
	Toluidine blue O	deep blue	+	+	+	+	+
Phenolic acids	Autofluorescence UV	light blue	+	+	-	+	+
Flavonoids	AlCl_3_ under UV	yellow	+	+	+	+	+
Pectins	Toluidine blue O	purple or pink	+	+	+	+	-

+ present, —absent, nd not determined.

**Table 2:** Multiple gradient development program in the analysis of flavonoids.

Elution Step	Development Distance [cm]	Mobile Phase Composition
1	3	6 mL solution A (toluene hexane: formic acid, 7:3: 0.1), 3 mL ethyl acetate, 1 mL methanol
2	9	5 mL solution A (toluene hexane: formic acid, 7:3: 0.1), 2.5 mL ethyl acetate, 2.5 mL methanol

**Table 3:** Multiple gradient development program in the analysis of phenolic acids.

Development Step	Mobile Phase Composition
1, 2	3 mL solution A (heptane: dichlorometane, 7:3), 2 mL diisopropyl ether, 0.1 mL 85% formic acid, 1 mL distilled water
3–7	4 mL solution A (heptane: dichlorometane, 7:3), 1 mL diisopropyl ether, 0.1 mL 85% formic acid

**Table 4:** Retention time in minutes and absorption maxima in the range 200–400 nm for phenolic acids in the HPLC method.

Phenolic Acid Standard	Retention Time [min]	λ_max_ [nm]
gallic acid	3.78	227.5; 271.0
protocatechuic acid	6.37	227.5; 258.6; 294.0
gentisic acid	12.23	234.9; 326.9
vanillic acid	14.05	226.4; 260.5; 291.0
caffeic acid	14.95	239.0; 322.6
syringic acid	16.85	224.7; 273.3
*p*-coumaric acid	26.12	228.7; 308.9
ferulic acid	31.93	234.9; 322.6
chlorogenic acid	41.75	234.9; 300.3

## Supplementary Materials

Figure S1: Flavonoid standards after visualization with aluminum chloride in VIS light; Table S1: Names of standards with CAS number, producer, and product number.
