# Supplementary material for: Phenolic Constituents of Lamium album L. subsp. album Flowers: Anatomical, Histochemical, and Phytochemical Study
Source: Molecules. 2020 Dec 19;25(24):6025. doi: 10.3390/molecules25246025 (PMC7766379; doi:10.3390/molecules25246025)
Supplement: Supplementary file 1 [file molecules-25-06025-s001.pdf]

Table S1. Names of standards with CAS number, producer and product number.

No	Name of standard	Producer	Product number	CAS number
1	acacetin	Sigma (USA)	49975	480-44-4
2	apigenin	Sigma (USA)	SMB00702	520-36-5
3	apigenin 7-glucoside	Supelco (USA)	44692	578-74-5
4	astragalin	Supelco (USA)	68437	480-10-4
5	hesperetin	Supelco (USA)	H4125	69097-99-0
6	hesperidin	Sigma (USA)	H5254	520-26-3
7	hyperoside	Sigma (USA)	00180585	482-36-0
8	isoquercitrin	Sigma (USA)	00140585	482-35-9
9	isoorientin	Supelco (USA)	78109	4261-42-1
10	kaempferol	Supelco (USA)	96353	520-18-3
11	quercetin	Sigma (USA)	Q4951	117-39-5
12	isorhamnetin	Sigma (USA)	17794	480-19-3
13	luteolin	Sigma (USA)	L9283	491-70-3
14	narcissin	Sigma (USA)	PHL83337	604-80-8
15	naringin	Sigma (USA)	N1376	10236-47-2
16	naringenin	Sigma (USA)	N5893	67604-48-2
17	isorhamnetin 3-gluco-7-rhamnoside	National Medicines Institute (Warsaw, Poland)		
18	rhoifolin	CarlRoth (Karlsruhe, Germany)	7420.2	17306-46-6
19	rutin	Sigma (USA)	R5143	207671-50-9
20	myricetin	Sigma (USA)	M6760	529-44-2
21	avicularin	Supelco (USA)	44006	572-30-5
22	isorhamnetin 3-glucoside	CarlRoth (Karlsruhe, Germany)	9386.2	5041-82-7
23	luteolin 7-glucoside	Supelco (USA)	49968	5373-11-5

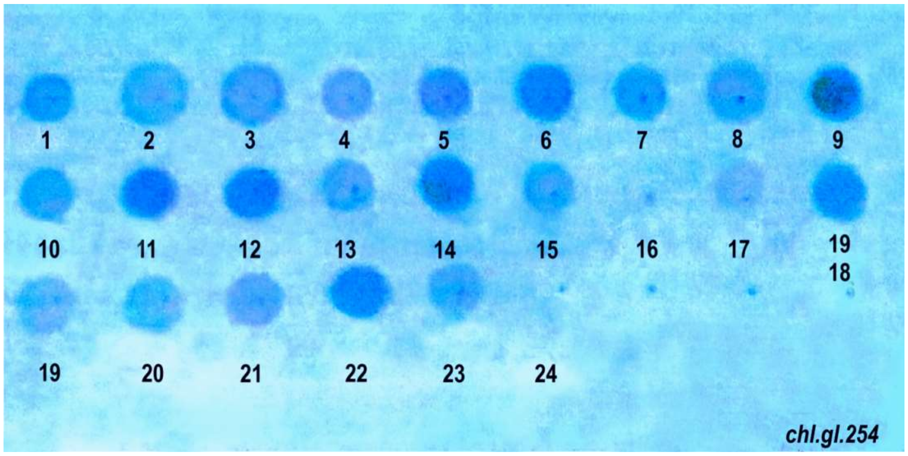

Figure S1. Flavonoid standards after visualization with aluminum chloride in VIS light. Names of standards: 1 - acacetin, 2 - apigenin, 3 - apigenin 7-glucoside, 4 - astragalin, 5 - hesperetin, 6 - hesperidin, 7 - hiperoside, 8 - isoquercitrin, 9 - isoorientin, 10 - kaempferol, 11 - quercetin, 12 - isorhamnetin, 13 - luteolin, 14 - narcissin, 15 - naringin, 16 - naringenin, 17 - isorhamnetin 3-gluco-7-rhamnoside, 18 - rhoifolin, 19 - rutin, 20 - myricetin, 21 - avicularin, 22 - isorhamnetin 3-glucoside, 23 -luteolin 7-glucoside.
